# Tobacco Mosaic Virus Movement: From Capsid Disassembly to Transport Through Plasmodesmata

**DOI:** 10.3390/v17020214

**Published:** 2025-01-31

**Authors:** Amr Ibrahim, Nobumitsu Sasaki, James E. Schoelz, Richard S. Nelson

**Affiliations:** 1 Department of Nucleic Acid and Protein Structure, Agricultural Genetic Engineering Research Institute, Agricultural Research Center, Giza 12619, Egypt; 2 Graduate School of Agriculture, Tokyo University of Agriculture and Technology (TUAT), Fuchu 183-8509, Japan; chaki@cc.tuat.ac.jp; 3 Division of Plant Science and Technology, University of Missouri, Columbia, MO 65211, USA; schoelzj@missouri.edu; 4 Independent Researcher, McKinney, TX 75071, USA; rsnelson1hq@outlook.com

**Keywords:** tobacco mosaic virus, virus movement, tobamoviruses, virus replication, plasmodesmata, movement protein, microfilaments, microtubules, vacuole, endoplasmic reticulum, plant-virus interaction

## Abstract

Determining mechanisms to establish an initial infection and form intracellular complexes for accumulation and movement of RNA plant viruses are important areas of study in plant virology. The impact of these findings on the basic understanding of plant molecular virology and its application in agriculture is significant. Studies with tobacco mosaic virus (TMV) and related tobamoviruses often provide important foundational knowledge for studies involving other viruses. Topics discussed here include capsid disassembly, establishment of a virus replication complex (VRC), and transport of the VRCs or virus components within the cell to locations at the plasmodesmata for intercellular virus RNA (vRNA) movement. Seminal findings with TMV and related tobamoviruses include detecting co-translational disassembly of the vRNA from the virus rod, full sequencing of genomic vRNA and production of infectious transcript for genetic studies determining virus components necessary for intercellular movement, and biochemical and cell biological studies determining the host factors, protein and membrane, needed for replication and movement. This review highlights many of the studies through the years on TMV and selected tobamoviruses that have impacted not only our understanding of tobamovirus accumulation and movement but also that of other plant viruses.

## 1. Introduction

**Figure 1:**
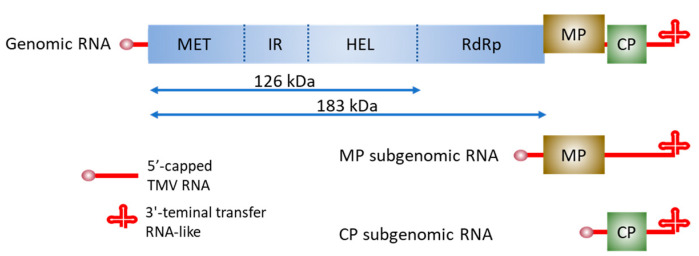
TMV genome and protein translation. The TMV RNA genome, which is 6395 nucleotides long, encodes, at minimum, four proteins required for normal plant infection: the 126 kDa protein, the 183 kDa protein, the movement protein (MP), and the capsid protein (CP) [4,6]. The replicase proteins (126 kDa and 183 kDa) are translated directly from the genomic RNA, whereas the MP and CP are translated from their respective subgenomic RNAs produced during TMV replication. The 183 kDa protein contains a methyltransferase domain (MET), an intervening non-conserved region (IR), an RNA helicase (HEL) domain, and an RNA-dependent RNA polymerase (RdRp) domain. The RdRp domain is expressed through a readthrough of an amber stop codon at the end of the 126 kDa protein open reading frame. Additional open reading frames are known and under study [13].

Tobacco mosaic virus (TMV), first shown to be a contagious agent by Mayer [1] and then characterized as filterable by Iwanovski [2], was named as a virus (originally defined as “contagium vivum fluidum”) by Beijerinck in 1898 [3]. For approximately 125 years since then, TMV has been a leading model virus in basic and applied virology research [4,5]. TMV was the first RNA virus to be completely sequenced, leading to the successful mapping of genes encoding four major proteins (126 kDa, 183 kDa, 30 kDa, and capsid protein (CP)) within the TMV genome [6] (Figure 1). This information, combined with the ability to mutate the sequence in full-length cDNA clones from which infectious transcript could be made [7,8], allowed TMV to be the first plant virus for which the functions of all the virus genes presumed essential for virus accumulation and spread were elucidated [4]. In addition to the CP, which is the structural component for virus RNA (vRNA) encapsidation, the 126 kDa and 183 kDa proteins are involved in virus replication and intercellular movement, and the 30 kDa protein, now usually referred to as the movement protein (MP), is essential for virus intercellular movement [4,9,10,11]. The CP is also essential for vascular-mediated infection [12]. Proteins from additional open reading frames (ORFs) within the genome were later identified and are being characterized [13].

Plant RNA viruses spread throughout the host by entry into a cell, translation of viral replication proteins, replication of parent vRNA, intra- and inter-cellular movement of virus components, and eventual vascular-mediated systemic movement [9,10,11]. Since viruses carry limited genetic information, they must successfully utilize intrinsic plant metabolic and macromolecule transport pathways to infect the host [14,15]. In this article, we focus on a critical review of significant findings in the literature describing how TMV moves intracellularly and intercellularly utilizing its RNA, replication proteins, and MP and their interactions with host factors. Capsid disassembly and aspects of virus replication are presented as they relate to intracellular movement. The literature is vast, and we do not pretend to include all relevant information, so we also reference other review articles with additional information on particular topics throughout this review. Virus entry into plant cells and environmental conditions that affect the establishment of infection are reviewed elsewhere [16,17,18,19,20,21]. Long-distance movement of TMV through the vasculature, the final step for systemic infection, likewise is reviewed elsewhere [22,23]. Through this review, we hope to provide insight into where further research should be directed to better elucidate the molecular mechanisms of TMV intracellular and intercellular spread.

## 2. TMV Capsid Disassembly and Aspects of Virus Replication Pertinent to TMV Movement

TMV has long been a model for basic research on virus disassembly and replication, with results providing important conceptual frameworks for research involving all viruses [24,25,26,27,28]. In this review, findings from tobamovirus TMV, the closely related tobamovirus species, tomato mosaic virus (ToMV, formerly TMV-L), and more distantly related tobamovirus species, obuda pepper virus (ObPV), youcai mosaic virus (YoMV), and turnip vein clearing virus (TVCV), are discussed [29]. TMV and ToMV retain close sequence identity (~80% or more) [30], whereas ObPV (formerly called the Ob-strain of TMV (e.g., [31,32]), YoMV [also called oilseed rape mosaic virus (ORMV) [33]] and TVCV [34] retain generally close genome organization and protein functions with TMV [13,29,35,36] (TMV genome organization—Figure 1). The latter two viruses represent multiple tobamovirus strains that infect brassica hosts [29,33,35,36].

### 2.1. Capsid Disassembly

**Figure 2:**
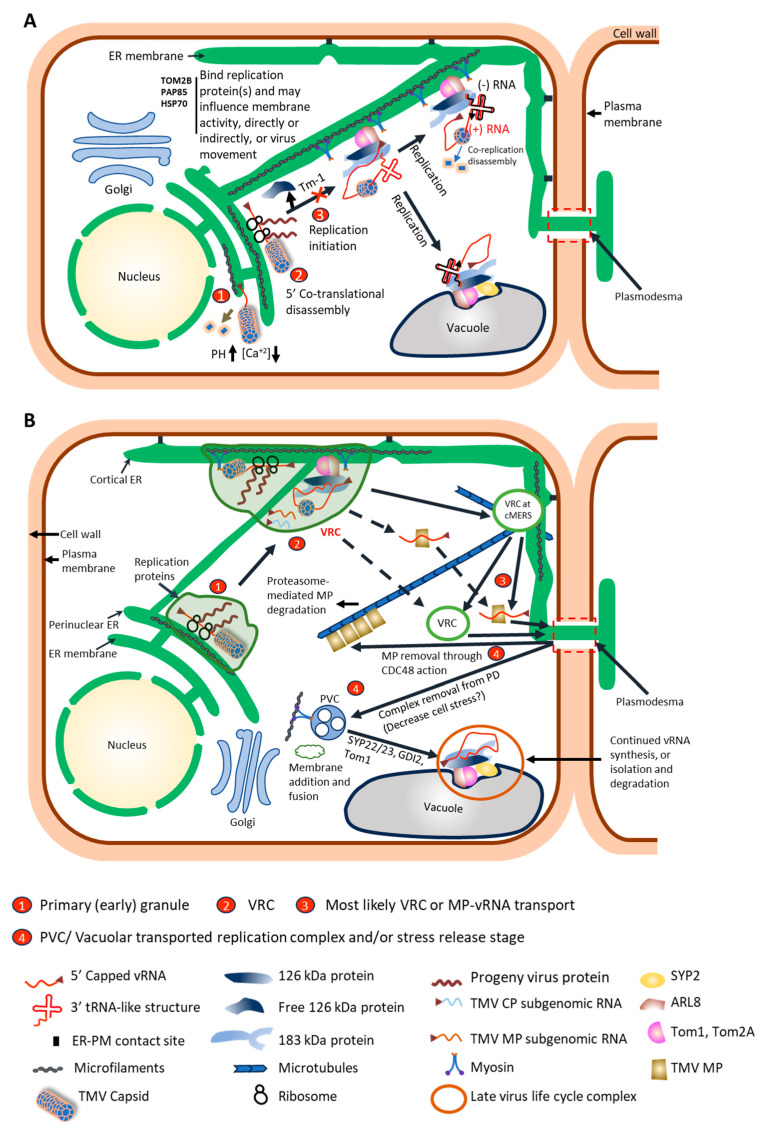
Models showing tobamovirus activity from rod disassembly to intracellular spread of TMV-induced complexes. (**A**) Formation of the virus replication complex and replication establishment. Once inside the cell, the virus rod composed of capsid protein (CP) monomers encounters higher pH and fewer calcium ions, weakening the structure of the virus rod, especially at the 5’ capped end of the viral RNA (vRNA) (step 1) [24]. The exposed 5’ end of the capped vRNA then associates with ribosomes and ER membrane. Co-translational disassembly proceeds, producing the 126 and 183 kDa proteins and removing 2/3 of the CP monomers beginning at the 5’ end of the vRNA (step 2) [38,39]. Translation is diminished, and replication and progeny vRNA 5’ end capping ensues as host factors Tobamovirus multiplication 1 (TOM1), Tobamovirus multiplication 2A (TOM2A), and ADP-ribosylation factor (ARF)-like small GTP-binding protein 8 (ARL8) associate with the 126 kDa protein, which binds the complex to membrane (likely ER origin, but other membranes possible) [50,51] and likely indirectly to the microfilament (MF) and myosin complex (step 3). Some 126 kDa protein does not associate with membrane and remains free in the cytoplasm to function as an RNA silencing suppressor [52,53,54]. Replication appears to require interaction of the 126 kDa protein, involving its methyltransferase domain-nonconserved intervening region, with the 5’ end of the vRNA, blocking further ribosome binding and translation [55], and the 126 kDa protein (involving the IR region) with the 3’ tRNA-like structure [56]. The interaction of the 126 kDa protein with the 3’ tRNA-like structure is either direct, involving the IR region, and/or indirect through 126 kDa protein interaction with eukaryotic elongation factor 1A (eEF1A), which also binds the 3’ tRNA-like structure (eEF1A not shown) [57]. The 183 kDa protein is required for replication, as it alone has the polymerase domain, and the 126 kDa protein and 183 kDa protein function in a 1:1 ratio [58]. Evidence exists that replication occurs at either or both the ER membrane and the tonoplast [28]. The *Tm-1* resistance gene functions to prevent host factors TOM1 and ARL8 from binding to form a competent replication complex (step 3) [50]. The 5’ capped RNA is also released with formation of full-length 126 kDa protein [55]. Minus (-) strand vRNA synthesis ensues, disassembling the remaining capsid protein monomers from the vRNA 3’-5’ (co-replication disassembly) [39]. Additional host factors are shown whose function in tobamovirus membrane-associated activities or movement are not fully understood [Tobamovirus multiplication 2B (TOM2B), vicilin-like seed storage protein (PAB85), heat shock protein 70 (HSP70)] [28,59,60]. (**B**) Intracellular spread of the TMV-induced complexes. A complex composed of the virus capsid with exposed 5’ capped vRNA attaches to ER, and translation begins (step 1) [42]. The complex is mobile, relying on MFs and associated host factors for movement within the cell, potentially from perinuclear area or other internal ER locations [42]. With time, viral movement protein (MP) and replication proteins, through their interaction with vRNA, host membrane (likely ER), and the actomyosin system, transport to the cortical region of the cell (step 2) [42,61,62]. Translation and replication continue in the complex, and the complex is referred to as the virus replication complex (VRC). These cortical granule complexes then associate with cortical microtubule (MT)-associated ER sites (cMERSs) at the MT, ER, and MF convergence with the plasma membrane [63]. From there the complex itself is transferred to the plasmodesma entrance, or the MP alone traffics vRNA to the plasmodesma entrance (step 3) [63]. In both cases, the MP drives association with the plasmodesma through its ability to direct itself with vRNA to that location. Other potential routes to the plasmodesma for the VRC or MP-vRNA complex are noted with dashed arrows. After intercellular movement is completed, there is a change in the complex in both location and content (step 4). The MP is extracted from the complex and positioned on MTs through the action of CELL-DIVISION-CYCLE protein48 (CDC48) [64], perhaps leading to proteasome-mediated MP degradation [65,66]. The remaining complex, now without MP, releases from the plasmodesma, and through interaction with membranes associated with prevacuolar compartments (PVCs) and/or vacuoles, transports away from the plasmodesma [67,68]. Transport is mediated through the actomyosin network. This transport may have multiple functions, such as removing stress in intercellular communications, thereby reducing a negative host response, to serving as a continuing replication site for genomic RNA or a storage site for spent virus protein, potentially leading to destruction of the protein [61,62,68,69,70,71]. Host factors such as syntaxin of plants 22 (SYP22), syntaxin of plants 23 (SYP23), RabGDP dissociation inhibitor 2 (GDI2), TOM1, and ARL8, all known to associate with prevacuolar or vacuolar membranes and the 126 kDa protein, would be associated with movement and function of these late virus life cycle complexes [51,67,72,73].

Disassembly of the TMV capsid is thought to be triggered by the higher pH and lower positively charged Ca^2+^ concentration in cells, leading to removal of a few CP subunits from the 5’ end of the viral RNA (vRNA; [37] and reviewed in [20,24]) (Figure 2A). Disassembly is considered to occur initially at the 5’ end of the RNA since interaction between CP and vRNA in this region is weak due to the absence of guanine residues (reviewed in [24]). Removal of CP continues from the 5’ end of the vRNA during translation to produce the viral 126 kDa and 183 kDa replication-associated proteins, a process referred to as co-translational disassembly [38,39]. Approximately 70% of the TMV genome is freed of the capsid in the 5’ to 3’ direction within 3 min of entry into a plant protoplast ([40], reviewed in [20]). The remaining 30% of the CPs at the 3’ end of the TMV vRNA is released by 30 min post cell entry [41]. It is believed that the CP associated with the 3’ end of the vRNA is removed as minus strand synthesis begins (i.e., co-replicational disassembly) since genomic vRNA encoding nonfunctional replication proteins did not uncoat at the 3’ end of the capsid while the addition of functional replication proteins *in trans* allowed uncoating [39]. Exactly how replication protein(s) and/or host factors remove the CP associated with the 3’ end of the vRNA is unknown. Parental vRNA, labeled prior to use with fluorescent Cy3-labeled UTP, is observed in small cytoplasmic granules within a minute of their injection into leaf trichome cells, and these granules form regardless of the presence of the CP ORF in the virus genome [42]. This indicates that the CP is not needed for the formation of the granule and that the granules are formed at the site of disassembly of the virus capsid during infection [42]. Granule formation did not require the presence of *cis* and *trans* elements necessary for virus replication (i.e., vRNA 3’ untranslated sequence or functional replicase proteins), although, without these elements, the granules were smaller and less stable [42]. The granules were shown to be associated with ER, and the 5’ methylguanosine cap on the vRNA was necessary to anchor the vRNA to the ER [42]. By 40 min post-inoculation, progeny virus particles appear in protoplasts [40].

Disassembly of the capsid in the first cell during infection is a unique process for TMV and other plant viruses in that, once inside the first cell, the virus is within the plant’s multicellular symplasm and able to move cell-to-cell without requiring some processes necessary to enter and disassemble in the first cell (e.g., the CP is not required to move between cells). However, once at the vascular system interface, additional virus protein (i.e., the CP) and host components are again required.

### 2.2. Formation of a Replication Complex and Genomic RNA Accumulation

Aspects of tobamovirus replication that involve plant membranes are emphasized in this section, as membrane association is often linked with the mobility of virus-induced intracellular complexes. Regarding replication, the TMV 126 kDa protein enhances it, and the 183 kDa protein is required for this activity [43,44]. The TMV 126 kDa protein, translated from the 5’-most vRNA ORF, has domains associated with methyltransferase and helicase activities [45,46] (Figure 1). The read-through 183 kDa protein includes an additional polymerase domain [47] (Figure 1). Steps in tobamovirus replication include the translation of the 5’ co-terminal 126 kDa and 183 kDa ORFs, translation-coupled binding of the replication proteins to the 5’-terminal region of the genomic RNA, recruitment of the genomic RNA replication protein complex onto membranes in association with host proteins, synthesis of complementary minus-strand RNA in the complex and synthesis of plus-strand subgenomic RNAs, one each for MP and CP synthesis, and genomic progeny RNA [28] (Figure 2A). Time points for critical steps in tobamovirus replication include the appearance of minus-strand vRNA accumulation at 2 h post-inoculation (hpi) and plus-strand appearance at no more than 4 hpi in protoplasts [48,49]. Plus-strand accumulation peaks at ~18 h post-inoculation [49].

To achieve TMV replication, the exposed 5’ methylguanosine cap on the parental vRNA is anchored to the ER, forming small granules [42]. These mobile granules require intact microfilaments (MFs) for movement [42]. This begins a close association of replication with membranes and the cytoskeleton during the entire process. This initial membrane association does not require active replicase and may occur through a host protein(s)-vRNA-mediated interaction [42]. Translation of the replication-associated proteins is concurrent or ensues.

It has been shown that the virus replication proteins first form a relatively unstable membrane association that becomes more stable upon interaction with membrane-associated host proteins, Tobamovirus multiplication 1 (TOM1), Tobamovirus multiplication 2A (TOM2A), and ADP-ribosylation factor (ARF)-like small GTP-binding protein (ARL8) [50]. These host proteins have been implicated in various functions associated with replication, including capping of the 5’ end [51,74,75] and the switch from translation to minus strand synthesis [28,51,72,76]. To initiate RNA replication, it is thought that the 126 kDa protein binds to the 5’ omega translation-enhancer sequence of the positive strand vRNA to inhibit further translation. On formation of the full-length 126 kDa protein and association with TOM1 and ARL8, further conformation alteration leads to the release of the 5’ omega sequence and binding of the vRNA 3’ UTR for replication [28,51,55]. This interaction may be influenced by the binding of replication proteins to eukaryotic elongation factor 1A (eEF1A), which itself binds to the viral 3’ UTR [57,77]. The TMV 126/183 kDa proteins themselves do not have obvious transmembrane domains, so their strong association with membranes is likely through interaction with these and other host proteins or through a predicted amphipathic helix [78], and further research is necessary to determine which, or if both, are required. Although the exact point where one may define an active virus replication complex (VRC) can be debated, it may be considered that the association of the host membrane proteins with replication proteins and initiation of minus-strand synthesis represents a VRC.

It is interesting that so many host factors, TOM1, TOM2A, ARL8, and syntaxin of plants 22 and 23 (SYP22 and SYP23), which interact, co-localize, or co-fractionate with the tobamoviral replication proteins and are necessary for normal virus accumulation, associate with or influence vacuolar or prevacuolar membranes and the associated compartments [51,67,72]. Nonetheless, it is the ER that is considered the site of TMV replication in many studies. This conclusion is supported by the fact that replication proteins directly or indirectly were shown to co-localize with ER [78,79]. Additionally, lysate from uninfected protoplasts that are treated to minimize the presence of vacuoles supported ToMV replication [80]. Also, there were additional unidentified membranes, possibly representing ER membrane, present in the co-fractionation studies of the replication and host membrane protein markers besides those associated with the vacuolar membrane [72]. Lastly, TMV infection induces ER aggregation early, which then goes back to the normal filamentous and tubular forms of ER later in infection [81]. The end of aggregation may represent the end of ER utilization for replication and intracellular movement activities.

An interesting characteristic of the tobamoviral 126 kDa protein is that while a portion of its pool in the cell is associated with membranes, a larger portion is present in the soluble fraction of a cell extract and is not associated with replication [72]. This soluble protein seems associated with the RNA silencing suppressor activity of the 126 kDa protein of TMV and ToMV [52,53,54]. The 126 kDa protein, or its homologs among tobamoviruses, binds to siRNA duplexes and likely sequesters them from interacting with the RNA-induced silencing complex [82,83,84]. Findings supporting this activity include the observation that an attenuated ToMV, L11, having debilitated suppressor activity, had less soluble 126/183 kDa protein relative to the membrane-bound protein [54]. Also, overexpression of the membrane-associated host protein, TOM1, that binds the 126 kDa protein, weakened RNA silencing suppression [54]. Although this review will not report on host resistance factors (e.g., NBS/LRR- or RNAi-based) and their associated virus factors generally (see [85,86] for such information), we will mention that host resistance factor, Tm-1, a 754-amino-acid protein that binds the replication-associated proteins, inhibits virus replication [87]. Mutations in the ToMV replicase proteins that affect this function map to the helicase domain [88,89]. Tm-1 alters the stability of the ToMV replication complex on membranes, but not the binding of the replication proteins to membrane surfaces or RNA synthesis of already formed complexes [50,87]. It appears that Tm-1 makes viral genomic RNA more susceptible to nucleases and prevents TOM1 and ARL8 interaction with the replication complex [50].

Lastly, regarding virus replication, there are host factors that interact with the replication proteins whose full mechanistic function(s) in virus accumulation are unknown. Here we will only note those associated with membrane interactions or virus movement. Tobamovirus multiplication 2B (TOM2B), a small basic protein, only when inhibited in expression with TOM2A, decreases ToMV accumulation [59]. Silencing the vicilin-like seed storage protein, PAP85, decreased TMV accumulation in protoplasts [60]. This protein is upregulated early in infection and associated with TMV-induced ER modification, likely in association with the 126-kDa protein [60], but nothing further is understood about its mode of action. The chaperone protein, heat shock protein 70 (Hsp70), also co-purifies with the replication proteins [28]. With other viruses, this protein family or those related proteins encoded by a virus itself are important for virus accumulation and spread [90].

## 3. Intracellular Movement to the Plasmodesmata

### 3.1. Virus Components for Intracellular Movement

The preceding overview of capsid disassembly and virus replication was important to allow a proper discussion of findings on the intracellular movement of the virus to the plasmodesmata (PD). For multiple viruses, there is data supporting the concept that virus replication and intracellular transport occur together in space and time (reviewed in [61,63,69,91,92]). The virus-encoded MP, which is not necessary for virus replication but is required for virus intercellular movement, is introduced in detail here, and its activity during intracellular movement of virus components is presented.

It is now accepted that plant viruses use an active mechanism to travel internally from their site of disassembly to the PD for movement to adjacent cells, and TMV has a central role in the development of models for virus intracellular movement. TMV was reported to move between cells in no more than 8 h in *N. tabacum* ([93]; reviewed in [94]). However, it may take less or more time for this movement to occur. Green fluorescent protein (GFP) expressed from the TMV or ToMV genome appears at approximately 2–4 h post-infection in cells adjacent to an initially infected cell (e.g., [95], [48]), while others observed movement after longer periods (17–36 hpi; [95,96]). The specific time of intercellular movement may depend on environmental and host conditions, whether movement from the first to the secondary cell or the secondary or tertiary cell is being observed (e.g., [95]), and the method used to observe the movement (e.g., monitoring free GFP expressed from the virus genome, representing passive movement, versus presence of infectious virus, likely representing active movement). In the following paragraphs, the literature on TMV intracellular movement is summarized, and a model of intracellular transport is presented that reflects findings from these studies. For information on other viruses or additional interpretations of results for TMV intracellular transport, the reader should consult the many review articles listed in the paragraphs below or here [15,97,98,99,100,101,102].

Findings from early studies using immunolocalization against tobamoviral replication protein and light or electron microscopy suggested that intracellular complexes changed components or structure over time, with early structures being smoothly granular and later structures containing electron-dense rope-like structures. The complexes represented by early structures were named viroplasms, and the complexes represented by the later structures were named X-bodies [103]. The ER was shown to be associated with TMV-induced intracellular complexes [79]. Viral replication proteins and MP, indirectly or directly, were shown to co-localize with the ER [79]. Studies with ObPV demonstrated that near the infection front, green fluorescent bodies representing an MP-GFP fusion were often paired on either side of the cell wall in adjacent cells [32]. Later time-course studies using light and electron microscopy immunolocalization demonstrated the presence of paired, TMV-induced bodies near the infection front and showed that they contained both the viral replication-associated protein(s) and the MP [68]. PD were present between the paired bodies; however, because of the high number of PD present in cells, it was not clear if this association was fortuitous or functional. Later in infection, just four to six cells back from the infection front, the bodies were no longer paired, having moved from the cell wall and now containing very high amounts of replication-associated protein but no MP. For ObPV, a similar loss of MP in fluorescent bodies was observed in cells behind the infection front [32]. Kawakami and co-workers reported that fluorescent bodies containing MP-GFP aligned with PD were present at 18 hpi of *N. tabacum* leaves with TMV [95]. This was prior to or during initial intercellular movement in this study. An association of these bodies with the viral replication proteins was not demonstrated but assumed by findings in previous studies analyzing virus components in the TMV-induced intracellular bodies at a similar time of infection [95]. Tilsner and co-workers identified vRNA (likely progeny vRNA) in TMV-associated small cytoplasmic bodies that only appeared near the infection front (i.e., an early stage of infection) [104]. These bodies had similarity to those detected near the infection front by Szecsi and co-workers [68], thus correlating the presence of vRNA with the replication proteins and MP early in infection. Also, vRNA was identified at late stages of infection in large perinuclear bodies that were suggested to represent the late structure of virus-induced complexes (e.g., X-bodies or those without MP) [104].

While the presence of the viral replication proteins, MP, and vRNA at the periphery of the cell in a timeframe associated with intercellular movement has been demonstrated, how these components arrive and/or accumulate there is not fully understood. Both the replication proteins and the MP bind RNA, with the MP binding non-specifically [28,105,106]. It is possible that any one of these proteins, or perhaps some combination working together, could contribute to intracellular movement of the viral genomic RNA. However, there are findings that limit the possibilities. Only during infection, and then only toward the infection front, and never during ectopic expression, were the tobamoviral replication protein(s) clearly observed near PD in leaves [68,78,103,107,108,109]. Most of the ectopic studies identified cytoplasmic or perinuclear distribution of the 126 kDa protein and did not co-express and visualize known plasmodesmal markers to fully ascertain the replicase intracellular location (exception, [109], see below for discussion). These findings likely explain why an infectious virus containing only replication protein ORFs does not move intercellularly ([33]; data not shown within [110]). However, although the viral replication proteins cannot move intercellularly when expressed alone, they (either the 126 kDa or 183 kDa proteins or both) were required for the intercellular spread of the virus as determined through genetic studies with mutant TMVs [111]. Interaction between the shared non-conserved region and helicase domain in the 126 kDa/183 kDa proteins appears to be required for this activity [112]. The expression of the 183 kDa protein alone is sufficient for intercellular movement since a mutant virus preventing synthesis of the 126 kDa protein still allowed intercellular movement [44]. Although findings from these studies indicate that the 126/183 kDa proteins affected intercellular movement, it is also possible that their role could be associated with intracellular movement, since reductions in the rate of intracellular movement could also ultimately reduce the rate of intercellular movement. It is significant to note that ectopically expressed 126 kDa protein fused with GFP forms a microfilament (MF)-dependent mobile intracellular complex, which may be needed ultimately for virus intercellular movement [107,108].

The role of the MP in intracellular spread seems less mysterious since it, when expressed alone, can traffic itself and RNA to the PD [113,114,115]. Interestingly, co-expression of the ToMV 126 kDa protein-GFP and MP-mCherry resulted in more numerous and larger 126 kDa protein intracellular bodies that contained MP-mCherry that, in some instances, were mobile [109]. Also, some smaller 126 kDa protein bodies became associated with the ends of filamentous MP-mCherry structures, suggesting a potential synergy provided by the MP. Genetic studies, however, do not support a direct interaction between the replication proteins and the MP [112].

With this information and that on capsid disassembly and genomic vRNA accumulation, an updated model of the intracellular trafficking of TMV components and their interaction with host factors can be described (Figure 2B). The reader should note, however, that there are other interpretations of results that would affect this model, and we will present some of the alternative interpretations. In the updated model proposed here, intracellular spread of TMV begins before virus replication, by association of 5’ capped parental vRNA with the ER to form granules [42]. The 5’ cap is required to anchor the vRNA to the ER [42]. The translation of the replication proteins ensues in these granules, and their association with the vRNA is modified as their 3-dimensional folding and 126-183- and replication-hetero-protein interactions proceed. These granules are mobile [42]. As replication proceeds, the MP is translated from subgenomic vRNA (reviewed in [116,117]). The MP also associates with ER [79,81,118,119], now thought to occur at the cytoplasmic face of the ER through two hydrophobic regions in the MP that are not transmembrane domains [120]. The viral replication proteins co-localize with the ER either directly through interaction with host proteins and/or vRNA associated with ER [79,118].

As noted earlier, it is unclear whether the MP acts alone in binding progeny vRNA for transport in granules towards the PD through the cortical region or whether the entire complex, containing the replication proteins, the VRC, traffics to the cortical cell area at or near PD (Figure 2B). In the former case, trafficking to the PD would be directed through the MP since it alone appears to have the capacity to associate with the PD. In the latter case, the transport of the VRC may or may not initially be to the PD, but eventually, the MP would be required for transport of vRNA to and through the PD.

### 3.2. Host Components for Intracellular Movement

The influence of host components on the intracellular movement of the complexes with progeny vRNA will now be discussed in detail. As noted, both the replication proteins and MP have known interactions with the ER [78,79,114], and transport of the vRNA, either with MP or within a VRC, to the PD, is suggested to be by the ER and its associated cytoskeleton (Figure 2B). Support for this conclusion begins with the fact that the intracellular ER is in continuum with the ER membrane passing through the PD. Secondly, the ER allows movement of embedded proteins or associated vesicles [121,122,123]. Lastly, disruption of the ER network with a high concentration of brefeldin A (BFA) reduces trafficking of MP to PD [113]. Although vacuolar and pre-vacuolar complexes are also dynamic, they do not move between cells, nor has the MP ever been associated with vacuolar or pre-vacuolar complexes. Inhibition of ER to Golgi secretory transport through treatment with a low concentration (10 ug/mL) of brefeldin A (BFA) or overexpression of a dominant-negative GTPase, Sar1p, does not alter targeting of virus-expressed or ectopically-expressed, fluorescently-labeled MP to the PD [113,124,125,126]. Although the low concentration of BFA did influence the structure of the MP-GFP inclusions, the general conclusion is that transport of the vRNA to the cell cortical area does not utilize COPII-mediated transport. This conclusion is further supported by the finding that Sar1p does not affect sustained virus intercellular movement [126]. Also, the cortical MP particles of YoMV do not co-localize with the endosomal membrane marker, ARA7-RFP, indicating that the particles are not associated with the prevacuolar compartment/ multivesicular bodies [33,127,128].

#### 3.2.1. TMV and Cytoskeleton

MFs and myosins are closely aligned with the ER membrane, and multiple studies have demonstrated that TMV VRCs and ectopically expressed 126 kDa protein or MP co-align with MFs and are mobile (reviewed in [61,62]). At the earliest time point in infection, the granules containing fluorescent parental vRNA show two types of movement: longitudinal transport along large ER strands and more restricted movement in the cell cortical layer that was co-localized with labeled MFs [42]. This movement was diminished by treatment with latrunculin B (LatB) [42]. The movement of these granules would be associated with the replication proteins that are attached early in synthesis to the progeny viral RNA. These early granules are presumed to be the progenitor of later cortical granules. The presence of MP in the cortical granules was demonstrated for multiple tobamoviruses [33,114,129]. In addition, the cortical granules contain vRNA and replication protein [104,114,118]. These cortical granules are mobile and traffic while co-aligned with ER and MFs (e.g., [33,114]). Treatment of tissue with LatB inhibited the appearance of particles at the PD and their internal movement [113,114]. The immunolocalized paired bodies containing the replication proteins and MP by light microscopy and EM near the infection front would represent the final destination of these cortical granules near the PD after transport and replication in the next cell had occurred [68,95].

Myosins are integral to transport along MFs, and the suggestion that they influenced TMV intracellular movement came through work showing that 2,3-butanedione monoxime, a general myosin motor inhibitor, inhibited TMV intercellular spread [95]. Harries and co-workers determined through silencing specific myosin family members that myosin XI-2 and not XI-F was necessary for normal sustained spread of TMV [130]. Further studies using dominant-negative myosin constructs identified myosin XI-2 and XI-K, as well as myosin VIII members 1, 2, and B, as important for virus spread [108]. Inhibition of myosin XI-2 and XI-K activity caused the MP-GFP to excessively accumulate in intracellular aggregates correlated with observed modifications of the ER structure and dynamics and diminished the efficiency, but not the ability, of MP-GFP to traffic to the PD [108]. Additionally, the inactivation of myosin XI-2 disrupted the movement of a 126 kDa protein-GFP fusion along MFs and caused smaller and more numerous 126 kDa protein-GFP aggregates. The 126 kDa protein-GFP aggregates were in a perinuclear position, which was noted as consistent with the effects of the dominant-negative myosin XI-2 and XI-K on ER structure and dynamics [108].

The finding that myosin class VIII members were important for virus spread was a novel discovery, as previous studies had not observed an effect of myosins VIII-1 or -2 on either virus intercellular movement or MP targeting to PD [130,131]. Amari and co-workers showed that dominant-negative inhibition of class VIII-1, -2, and -B myosins reduced localization of transiently expressed MP-GFP at PD and, during inhibition of myosin VIII-B, supported its redistribution to the plasma membrane (PM) [108]. The importance of specific myosin XI and VIII family members for TMV intracellular spread was theorized to be for, respectively, concentrating VRCs to cortical microtubule (MT)-associated ER sites (cMERSs; reviewed in [132]) associated with MFs and adjacent to the PM at or near the PD and subsequent transport of MP with vRNA to the PD from this site.

An unusual finding at this time (~2010) requires extensive thought when considering the need for MFs during intracellular movement of TMV. Hofmann and co-workers determined that disruption of MFs with LatB did not disturb virus transport within the first 24 h after treatment [133]. Also, by transiently overexpressing actin-binding protein 2 (ABD2), which was hypothesized to freeze ER membrane fluid dynamics, they found that virus intercellular transport was reduced. From these two observations, the authors concluded that MFs were not necessary for short-term virus intercellular transport and hypothesized that continuing ER-embedded protein fluid dynamics were sufficient for the transport of vRNA and MP associated with the ER to the PD. ER remodeling is fairly quickly achieved by LatB treatment, but lateral diffusive flow of proteins in the ER, while slowed at various rates according to experimental procedure, was less affected in short periods [121,123]. Previous virus intercellular transport studies utilizing LatB or inhibiting myosin activity generally involved observations at 2 days post-treatment or more [95,108,130]. Support for ER-mediated lateral diffusion of virus components comes from the finding that TVCV intercellular movement was not inhibited by LatB treatments through to the end of the experiment at 6 dpi [130].

The concept that virus complex movement associated with ER may not be driven by a specific interaction with actin has been considered [69]. However, it remains a mystery how the rapid effects of myosin dominant-negative inhibition or LatB treatment on early granule and cortical granule intracellular movement, ectopically expressed TMV MP or 126 kDa intracellular movement, or MP targeting to PD (e.g., [107,108,113,114]), cannot have an effect on early virus intercellular movement. Hofmann and co-workers treated plants multiple days after infection, allowing a build-up in virus components pre-positioned for movement prior to treatment, and these components may have continued for a short term (24 h) to move in the ER between cells after LatB treatment [133]. Harries and co-workers treated plants with LatB prior to inoculation when they observed inhibited intercellular spread of TMV from 2 dpi forward [130]. Thus, perhaps the simplest interpretation is that ER-mediated transport within a cell is critical for short-term virus intracellular transport, but that the actomyosin system affects ER function over longer periods, modulating multiple steps in intracellular vRNA and virus component movement that, together, become measured in reduced intercellular spread over a longer period. Supportive of the influence of MFs in such a model is that overexpression of ABD2, which was considered to freeze ER membrane protein transport, inhibited TMV spread [133]. The lack of effect of LatB on TVCV intercellular spread over long periods observed by Harries and coworkers, however, may suggest that a major revision of how tobamoviruses utilize the actomyosin network for spread is needed [130].

Regarding MTs, early studies identified an interaction or co-localization between the TMV MP and MTs, and it was suggested that MTs were utilized for the transport of MP and vRNA to the PD [134,135]. Later studies found that manipulation of MT organization or the ability of MPs to associate with MTs could influence virus intercellular movement (e.g., [129,136,137]). However, several studies using pharmacological disruption of MTs saw no effect on intracellular transport of MP, accumulation of MP in PD, or virus intercellular movement, suggesting that intracellular virus transport was unaffected [66,95,113]. It was noted that pharmacological treatments do not always disrupt all MT arrays [138]; however, silencing alpha-tubulin in place of a pharmacological treatment also did not alter virus transport [66].

Studies of interaction between MTs and MPs or VRCs were later focused on two areas. Firstly, studies showed that VRCs were localized to cMERSs ([129]; reviewed in [132]). It was proposed that tobamoviral MP-tagged VRCs visualized as the small cortical particles become attached at cMERSs and then the MP with vRNA or possibly the entire VRC detaches and moves to the PD, directed by the MP [33,63,139]. Movement may be driven more by the ER/actomyosin complex than MTs, although it may be aligned with MTs. As such, MTs would have more impact on the localization of virus complexes and thus an indirect impact on the active virus component involved in intracellular transport to the PD. It will be important to determine the stability of the MTs in cMERSs during pharmacological treatment or silencing of alpha-tubulin expression in order to clarify MT function during TMV intracellular movement.

The second area of interest in TMV interaction with MTs was the association of the MP with MTs late in infection [140]. An elegant study demonstrated that the TMV MP is extracted from membranes by CELL-DIVISION-CYCLE protein48 (CDC48) through the MP N-terminus to the cytosol, where the MP subsequently attaches to MTs [64]. This finding may explain the relatively sudden loss of MP in the virus inclusions containing replication proteins observed just six cells behind the TMV infection front in leaf cells [68]. It was later shown through studies with the MP of YoMV that this association with MTs was not required for virus movement [33]. A similar finding was reported with the MP of TVCV and a mutant MP of TMV [34,66]. Thus, MT impact on intracellular movement may be more towards (a) providing a location to organize virus accumulation/spread rather than actively aiding spread itself and (b) for late interaction with MP after virus spread has occurred (see Section 4.6 for proposed function of this late interaction).

#### 3.2.2. TMV and Prevacuolar Compartment/Vacuole Association

The literature review and modeling to this point have addressed many issues regarding membrane and cytoskeletal activities necessary for intracellular movement of TMV/tobamovirus components. However, one area that has not been reviewed in detail in TMV literature is the interesting finding that many of the host proteins shown to interact with the TMV replication protein(s) are associated with or influence the vacuole or pre-vacuolar compartments, specifically involving their membranes. As previously noted, it has been determined that the replication proteins and replicase activity of ToMV were associated primarily with the vacuolar membrane, likely through interaction with host proteins TOM1 and TOM2A [72]. Also, ARL8 was found to interact with the replication proteins [51]. This protein is associated with vacuolar membranes as well as in the PD [141,142]. RabGDP dissociation inhibitor 2 (GDI2), which interacts with the TMV 126 kDa protein [73], is important for vesicle trafficking pathways. Silencing this protein induced vesicles associated with a vacuolar and prevacuolar compartment membrane marker, Vam3 (also known as Qa-SNARE SYP22), that appeared similar to those induced in TMV-infected transgenic plants expressing the same SYP22. Recently SYP22 itself and SYP23 were shown to interact with the TMV 126 kDa protein and to be necessary for normal virus intercellular movement and accumulation [67]. Interestingly, the 126 kDa protein was shown to co-localize with the exterior of the vacuolar membrane decorated with mRFP-SYP22 in a 3-dimensional rendering [67]. This finding suggests that re-localization of these vacuole- and prevacuole-associated membrane proteins to other membranes to support tobamovirus infection is not occurring, but further work with the other host proteins and during virus infection is necessary to support this conclusion. Decreased expression of these vacuole- and prevacuole-associated proteins affects virus physiology in various ways, either diminishing virus accumulation and/or virus spread [51,59,67,141], but in the case of GDI2, increasing susceptibility to infection [73]. It is possible that TMV can utilize both the ER and the vacuolar membranes simultaneously for accumulation, but the potential also exists that vRNA accumulation associated with these membranes occurs in series; first associated with the ER and then with vacuole pathway membrane constituents (Figure 2B).

To further define this thought, SYP22 is a Qa-SNARE protein associated with membrane fusions, likely between the prevacuolar compartments/multivesicular bodies and tonoplast (reviewed in [142,143]). The MP has not been shown to associate with vacuolar components. Thus, perhaps after the VRC has completed its function in intracellular movement, it releases from the plasmodesmal area, associated with the release of the MP to MTs mediated by CDC48, and proceeds internally for further replication or some other activity (Figure 2B). Support for this concept comes from the time course study by Szecsi and co-workers showing that the complexes containing replication protein move from the cell wall near the infection front to the interior of the cell late in infection [68]. The concept that regulation of virus protein or complex accumulation during infection enables maximum virus accumulation, movement, host survival, and/or avoidance of plant defenses has been presented (e.g., [69,70,71]). Liu and Nelson suggested that the removal of the TMV VRC from the plasmodesmal area may relieve a cellular stress response that would limit virus spread [61]. Results from the Culver laboratory indicate that loss of ATPase activity (necessary for helicase activity) in a mutant 126 kDa protein causes this protein to form large granular inclusions containing tubule-like inclusions similar to those found in X-bodies behind the infection front as opposed to small inclusions formed by a native 126 kDa protein similar to VRCs observed at the infection front [71]. Due to the loss of ATPase activity in the mutant 126 kDa protein, they speculated that the large inclusions could represent aggregation of “spent” replication protein [71], which could be subject to degradation [70]. Others suggested that with the completion of the intercellular movement, the expression of the MP is no longer needed, and the VRC is transported and transformed into a singular replication factory [62,101]. Some of these possibilities are not mutually exclusive. Knowing that ARL8 protein is associated with both the vacuole and PD and that SYP2 proteins are important for fusing membranes, the potential that membrane recycling from the plasmodesmal area, potentially as vesicles toward the center of the cell, is important to consider. Additionally, considering that YoMV MP did not co-localize with ARA7 (a canonical RAB5) [33] while the 126 kDa protein interacts with SYP22, which associates with a tethering complex associated with RAB5 activity and vacuolar membranes [144,145], there is further evidence of a divergence in virus complex membrane association with the departure of the MP. This membrane recycling could continue to create an environment with high levels of membrane to allow enhanced virus replication. The viral replicase was shown to be active in the vacuole membrane-enhanced cell extract fractions [72]. Such membrane buildup using the actin highway has been postulated for tomato bushy stunt virus accumulation (e.g., [130,146,147]). Vesicular movement internally associated with the actomyosin network would not affect virus intercellular movement directly, as movement would have already occurred. This would fit with the observation that actomyosin is required for sustained virus movement but not for immediate movement. Additionally, it could help explain why TVCV, which does not produce large visible intracellular complexes during ectopic expression of its 126 kDa protein homolog [130], does not require actin or myosin XI-2 for its sustained spread [130]. Niehl and coworkers determined that the MP of ORMV, which is in the same tobamovirus subgroup as TVCV, is not associated with as large fluorescent structures as the MP from TMV during virus infection [33]. It will be worthwhile to observe infectious constructs of chimeric TMV and TVCV or ORMV with opposing replication proteins to determine their interaction with these vacuolar or prevacuolar membrane-associated proteins and susceptibility to actomyosin inhibitors. However, even if there is a divergence between TMV and TVCV/ORMV in their MF requirements for post-PD localization, there remains a question of how TVCV/ORMV complexes move to the PD without MFs. There remains much work to be conducted to understand tobamovirus intracellular transport.

## 4. Intercellular Movement of TMV

### 4.1. MP-Facilitated TMV Intercellular Movement

Plant cells are interconnected by the membranous channels, PD, which traverse their cell walls and function in cell-to-cell communication (for review see [148,149]). Numerous PD can exist in a cell wall, and they can cluster in groups referred to as pitfields. PD are classified into primary PD which form during cytokinesis and secondary PD which form in the cell walls of non-dividing cells. A simple depiction of a plasmodesma is a pore in the cell walls of two adjacent cells, lined with PM and containing a concentric ER tube known as the desmotubule. The desmotubule connects the ER networks of both cells and delimits with the PM a cytosolic area, known as the cytoplasmic sleeve, involved in symplasmic transport. The size of molecules that can go through the plasmodesma, i.e., its size exclusion limit (SEL), was originally presumed dependent on the size of the cytoplasmic sleeve. Molecules below the SEL can passively diffuse through, while molecules above the SEL require active transport involving the dilation of PD to accommodate their trafficking [150]. The main route for transferring molecules through PD is through the cytoplasmic sleeve. However, the translocation of small molecules, up to 10.4 kDa in size, through PD via the ER lumen has been reported [151]. Also, the transport of proteins attached to the desmotubule membrane occurs [110].

The SEL of PD is tightly regulated and responds to changes during development and in response to stresses. For many years, the main mechanism for regulating PD permeability was attributed to callose deposition and degradation in areas around PD openings (neck regions) in closing and opening PD, respectively. However, the close proximity of the two membranes, desmotubule and PM, along with the presence of known membrane contact site proteins in PD, such as vesicle-associated membrane protein-associated proteins (VAPs), actin-binding protein networked (NET) 3C, multiple C2 domains and transmembrane region proteins (MCTPs), and synaptotagmins (SYTs), identify PD as specialized membrane contact sites that could influence PD permeability [91,152]. Ultrastructure analysis using electron tomography of PD confirmed the remodeling of ER-PM contact sites (EPCSs) within PD during development and distinguished two morphological types of PD [153]. Type I PD are present in post-cytokinesis walls and are characterized by a close association of desmotubules and PM with no apparent cytoplasmic sleeves, whereas Type II PD in older tissues have recognizable cytoplasmic sleeves with spoke-like tethers bridging the desmotubule and PM and exhibit the typical PD structure. Interestingly, Type I PD lacking the cytoplasmic sleeves were found to support the movement of GFP; however, the mechanism is not clear [153]. Moreover, the MCTPs were shown to bridge the desmotubule membrane and the adjacent PM, suggesting their ability to bring the two membranes together and regulate PD permeability [154].

In addition to being a conduit for trafficking cellular constituents, PD are the routes that plant viruses and other pathogens use to spread their infection [92,149]. The intercellular movement of TMV from infected to uninfected cells through PD requires the combined actions of its MP and replication protein(s) [111,155,156]. TMV infection likely spreads through PD as a viral ribonucleoprotein (vRNP) complex containing, among other components, MP and TMV RNA [157], and not as virions, since CP is not required for intercellular movement [158]. Supporting this conclusion is the confirmed role of TMV MP to cooperatively bind, albeit non-specifically, single-stranded RNAs in vitro and associate with and traffic through PD in infected plants and when ectopically expressed alone [159]. Moreover, TMV MP can transport its mRNA through PD [160]. The intercellular spread of TMV requires the MP to target and increase the SEL of the PD to accommodate the passage of the vRNPs [161]. The TMV MP localizes mainly to the central cavity of the PD without an apparent presence at the plasmodesmal neck regions. This localization pattern was demonstrated by immunogold labeling and electron microscopy on tissues infected with wild-type virus [162] and confocal microscopy using tissues infected with virus expressing MP fused to GFP [163]. In addition, transgenic tobacco lines expressing TMV MP showed MP preferential accumulation inside PD [164]. The MP-preferred PD type in these plants was branched secondary PD. The association of TMV MP with primary PD in immature tissues of tobacco sink leaves, Arabidopsis embryos, and just above the root meristem of young Arabidopsis seedlings has now been reported [165].

### 4.2. MP Targeting to Plasmodesmata

To understand TMV MP targeting to the PD, research was conducted to identify PD targeting signal(s) within the MP amino acid sequence. First, the N-terminal 50 amino acids of the MP were found to include a major plasmodesmal localization signal (PLS) that is both necessary and sufficient for PD targeting [166]. Mutational analysis of this PLS sequence revealed that Val-4 and Phe-14 amino acid residues are essential for the proper targeting of MP to the PD [166]. Later, two additional PLS domains, including amino acid residues 61–80 and 147–170, were shown to have PD targeting capability [167]. Interestingly, the major PLS and the two additional PLS domains were sufficient, when fused individually, to target a cargo protein, cyan fluorescent protein, to the outside openings of the PD but not for entering them. Therefore, these domains cannot mediate transport through the PD channels on their own. The C-terminal 16 amino acids of TMV MP also affect the subcellular location of TMV MP. Previously, it was determined that deleting 9 or 33 amino acids from the TMV MP C-terminus did not affect TMV intercellular spread [168]. Later it was determined that deleting the C-terminal 16 amino acids, while still allowing intercellular MP movement, localized the MP not only to PD but to the PM, in contrast to the unmutated MP, which accumulated only at the PD [169].

The mechanism by which the MP PLS sequences facilitated PD targeting was previously unknown, but we may now have a clue. Previously it was determined that the Arabidopsis host factor synaptotagmin A (SYTA, also known as SYT1) interacts with TMV and TVCV MPs and was necessary for normal intercellular transport [170,171]. SYTA is a member of the Arabidopsis SYT family of modular proteins that is involved in tethering the ER and the PM in EPCSs [172]. In addition, findings in an early report suggested that SYTA functions in the regulation of endocytosis and endosome recycling at PM [170]. SYTA is anchored to the ER membrane through a N-terminal transmembrane domain and tethers the PM to the ER membrane through C-terminus Ca^2+^- and phospholipid-binding C2 domains [172]. Evidence supporting this tethering activity comes from the finding that the cortical ER reticulate structure, which results from ER attachments to the PM at EPCSs, is disrupted in *syta-1* knockdown mutant plants [171]. By contrast, the actin cytoskeleton, known for organizing and stabilizing the ER, retains its structure in these mutant plants [171]. In addition to the TM and two C2 domains (C2A and C2B), SYTA includes a synaptotagmin-like mitochondrial-lipid-binding protein domain involved in lipid exchange between the ER membrane and the PM [172]. In subcellular localization studies, SYTA was partially detected around and within the PD [171,173,174]. The targeting of TMV [175] and TVCV [171] MPs to PD was reduced in *syta-1* plants compared to wild-type plants. Interestingly, the PLS sequences of the TMV MP, including amino acids 1–50 and 61–80, were shown to interact with SYTA using a yeast two-hybrid assay [167,175]. Furthermore, TMV MP or its major PLS domain (MP^1–50^) interacted with SYTA at the PM EPCSs in BiFC assays [175]. The PLS sequence was necessary and sufficient for this interaction [175]. Similar to the full-length TMV MP, the accumulation of PLS fused to cyan fluorescent protein at PD was greatly reduced in the *syta-1* mutant compared to wild-type plants, suggesting that the PLS-SYTA interaction is crucially important for targeting the MP to the PD [175]. In addition, the TVCV MP was found to interact with SYTA *in planta* using a bimolecular fluorescence complementation (BiFC) assay and recruit it to the PD during early infection [171]. In uninfected cells, ectopically expressed SYTA and TVCV MP failed to show an interaction in the BiFC assay, as SYTA localized to EPCSs near PD, where the TVCV MP was present [171]. This suggests that the interaction is transient in the absence of TVCV infection [171].

**Figure 3:**
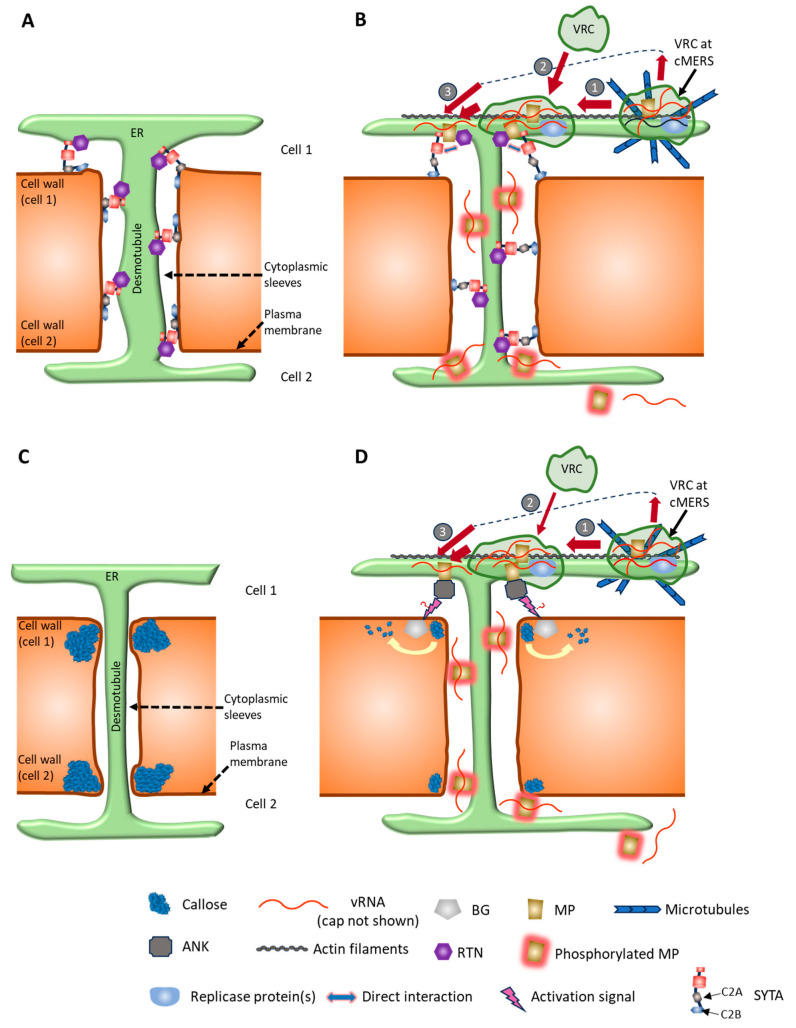
A model for modulating plasmodesmata (PD) permeability for tobacco mosaic virus (TMV) intercellular movement. TMV movement protein (MP) is shown to modify PD permeability by interacting in at least two activities: changing PD membrane morphology through the tethering function of the ER-plasma membrane (PM) contact site (EPCS) proteins like synaptotagmin A (SYTA) (panels **A**,**B**) and callose accumulation and degradation at PD neck regions (**C**,**D**). Actions of TMV MP in these two activities are presented separately for figure simplification and because it is not clear if these two activities work together or independently, in parallel or in series, for TMV intercellular transport. Findings from a recent study show that in the absence of multiple C2 domains and transmembrane region proteins (MCTPs), representing EPCS proteins, PD trafficking is insensitive to callose accumulation or degradation [181]. (**A**) Model for EPCS-based limited PD permeability. EPCSs at PD entrances contain SYTA, SYT5, and SYT7 tethering proteins [174]. In addition, SYTA is suggested to be inside the PD [171,173,174] and can interact with itself, SYT5, and SYT7 [174] and form a complex (only SYTA is shown for clarity in the figure). SYTA and SYT7 also interact with reticulin 3 (RTN 3) and 6 at PD [182]. SYT proteins can form complexes through their synaptotagmin-like mitochondrial-lipid-binding domain interactions. SYTA is inserted into the ER membrane through its N-terminus transmembrane domain and docks with PM through its C2B domain, which constitutively associates with PM [173]. The SYT proteins work with RTNs to close PD by bringing the ER membrane at the plasmodesmal entrance and the desmotubule into close apposition to the PM, thereby reducing the plasmodesmal orifice and cytoplasmic sleeve sizes. It was proposed that this activity is regulated by stress-induced Ca^+2^ concentration levels [172]. Ca^+2^ concentration increases in addition to local accumulation of anionic phospholipids at PM, leading to docking of the C2A domain with the cytosolic leaflet of the PM and decreased distance between ER and PM [172]. (**B**) In TMV-infected cells, virus replication complexes (VRCs), including MP, can arrive close to PD openings at cortical ER regions [e.g., cortical microtubule (MT)-associated ER sites (cMERSs)] and move in an actinomyosin-dependent fashion to EPCSs at PD (step 1) or move directly from other cortical ER regions to these EPCSs (step 2). As MP can bind its RNA and target it to PD in the absence of other TMV components, it is assumed that MP binds vRNA and transports it from VRCs at any location, as vRNP complexes, to EPCSs at PD (step 3). However, MP only can bind SYTA and localize to the EPCSs. MP interactions with SYTA and RTNs can interfere with or disrupt the docking of the C2A domains to the PM, hence increasing the distance between the ER membrane and desmotubule to the PM, allowing the transport of vRNP complexes. (**C**) Model illustrating limited PD permeability due to callose accumulation at the cell wall around plasmodesma neck. Callose accumulation results in the cell wall pushing the PM against the ER-based desmotubule and decreases PD intercellular trafficking. (**D**) Model illustrating an increase in plasmodesmal SEL through TMV MP-mediated callose degradation. MP can arrive at EPCSs at PD as described in (**B**). At PD, TMV MP can interact with ankyrin repeat-containing protein (ANK), and this interaction might activate or recruit a BG protein through an unknown mechanism, which can catalyze callose degradation and increase PD opening [183]. Within the PD, MP can be phosphorylated by a plasmodesmal protein kinase (not shown) during its passage through PD to allow translation in the next cell (see text for further description). Aside from MP likely trafficking vRNA, other viral or cellular components of TMV mobile vRNPs and the mechanism underlying their transport through PD are not fully understood. Diffusion of these vRNPs in association with the desmotubule membrane across PD has been proposed [94]. The illustrated protein-protein interactions may not accurately show the protein domains involved.

As indicated, the targeting of TVCV MP to PD was reduced in *syta-1* plants [171]. This reduction was not a general effect on host secretory trafficking, as the secretory-dependent targeting of PDLP1-GFP to PD remained unchanged in the mutant plants [171]. Taken together, the findings that TVCV MP interacts with SYTA at EPCSs near the PD and recruits it to the PD in early infected cells where MP-mediated intercellular transport occurs, that TVCV VRCs formed in *syta-1* plants are substantially smaller than those in wild-type plants, and that the absence of SYTA inhibits the targeting of MP to the PD suggest that the MP-SYTA interaction could result in the remodeling of EPCSs to establish TVCV VRCs containing SYTA and MP at the PD and in relocating SYTA within the PD [171]. This process was hypothesized to regulate PD permeability for MP-based viral intercellular movement and to link TVCV replication and movement [171], as has been suggested for other viruses [91]. Further work is needed to elucidate the mechanism of SYTA-based regulation of plasmodesmal SEL through MP interaction (for one model, see Figure 3A,B).

In a recent study aimed at understanding the molecular basis of the resistance gene *Tm-2^2^* and its conferred protection against TMV and other tobamovirus infections, a conserved cysteine (C68) in the TMV MP sequence was identified as playing a critical role in regulating TMV movement and triggering *Tm-2^2^* resistance [176]. The C68 present in the TMV MP region earlier was determined to be important for MP folding [106] and association with ER [120]. Modeling and analyzing the 3-D structure of the MPs of TMV, cauliflower mosaic virus, cucumber mosaic virus (CMV), tomato bushy stunt virus, and tomato spotted wilt virus using AlphaFold2 showed that the C68 residue resides in a conserved putative jelly-roll fold in these MPs [176]. Substituting C68 with histidine compromised TMV MP function by decreasing its association with ER and PD, increasing accumulation in immobile bodies, increasing miss-targeting to the PM, and abolishing its intercellular movement activity. These observations suggest that C68 is required for the proper interaction of MP with ER, which is required for the PD targeting of TMV MP [176].

Regarding other host-MP interactions, the receptor-like kinase BARELY ANY MERISTEM 1 (BAM1) interacts with TMV MP *in planta* and affects its targeting to the PD [177]. BAM1 functions to facilitate intercellular siRNA transport and localizes to the PM and PD [178]. TMV MP interacts with both *A. thaliana*- and *N. benthamiana*-BAM1 [177]. Domain deletion analysis of BAM1 showed that the transmembrane and/or kinase catalytic domains of BAM1 are responsible for the interaction with TMV MP and suggested a role of the TM domain in localizing the MP-BAM1 complex to the PD [177]. The intercellular movement of TMV MP, as well as the local spread of TMV, was reduced in BAM1-silenced *N. benthamiana* plants [177]. Similarly, a putative transcriptional coactivator, KELP, was reported to interact with ToMV MP in vitro [179]. KELP overexpression did not affect ToMV replication in protoplasts; however, it restricted ToMV intercellular movement in *N. benthamiana* leaves. Additionally, KELP overexpression relocated ToMV MP, which associates with PD when expressed alone, to the nucleus and cytosolic filamentous or irregular structures, significantly decreasing its association with PD [180].

### 4.3. MP and Modulation of PD Permeability

Transport of TMV vRNP complexes is correlated with the action of the MP to increase the plasmodesmal SEL: a process termed PD gating. At the leading edge of an expanding infection on a leaf, the MP of TMV localizes to the center of the PD and increases plasmodesmal SEL. The increase in plasmodesmal SEL is restricted to cells at the leading edge of infection, as cells behind the leading front revert to a normal plasmodesmal SEL despite the continuous association of MP with the PD in these cells [163]. Significant progress has been made in understanding how TMV MP modulates the PD gating. Previously it was determined that TMV infection was modulated by deposition of callose, a β-1,3-glucan polymer, around the openings of PD [92,150]. Callose turnover (i.e., synthesis and degradation) is a tightly controlled cellular process involved in regulating the intercellular transport of different macromolecules, e.g., RNAs and proteins, essential for plant growth, development, and responses to biotic and abiotic stimuli [184] (Figure 3C,D). The deposition of callose at the cell-wall-constricted PD neck regions acts as a sphincter to limit trafficking through the PD. In contrast, callose degradation increases PD permeability and enhances intercellular transport. Callose synthesis and degradation at PD neck regions are mediated by the activities of the callose synthase, otherwise known as glucan synthase-like, and the β-1,3 glucanase (BG) families of enzymes, respectively [185]. Knocking down expression of a tobacco BG, GLU I, or knocking out expression of PD-associated Arabidopsis BG, AtBG_pap (plasmodesmal-associated protein), results in increased callose accumulation at PD, decreased PD permeability, and reduced, respectively, TMV or TVCV intercellular movement [186,187]. Overexpression of tobacco GLU I encoded by a TMV vector resulted in enhanced TMV intercellular spread [188]. A putative link between TMV MP function and callose-mediated virus spread has been identified [183]. TMV MP likely interacts with a tobacco cytoplasmic ankyrin repeat-containing protein at the PD, and this interaction is associated with callose reduction at the PD and increased MP and TMV intercellular spread (Figure 3C,D). Further supporting the role of callose in mediating virus intercellular movement, mutant plants for a callose synthase 8, *cals8-1*, or PDLP5, *pdlp5-1*, showing significant reduction in callose accumulation at PD, allowed enhanced TMV MP intercellular movement [189,190]. PDLP5 has recently been determined to interact with the PD-enriched t18:0-based sphingolipid species to regulate PD permeability by modulating callose deposition at PD neck regions [191].

In a recent study, TMV MP was found to interact with *N. benthamiana* class 1 reversibly glycosylated polypeptide (RGP), NbRGP1, in vitro and in vivo [192]. RGPs are known to function in cell wall biosynthesis and localize to the Golgi, but examples of localization to both the Golgi and PD have also been reported [92]. Transiently overexpressing class 1 NbRGP1, 2, or 3 in *N. benthamiana* leaves resulted in reduced TMV-GFP accumulation and intercellular movement [192]. Overexpression of NbRGP1 or 2 was associated with increased callose deposition at PD and was suggested to cause the reduced TMV-GFP movement observed. Overexpression of NbRGP3, however, decreased callose deposition at PD, raising the possibility that NbRGP3’s role in callose accumulation either requires the formation of a complex with other NbRGPs or is affected by its spatio-temporal expression profile [192]. These results generally confirmed an earlier finding that expressing an Arabidopsis RGP gene fused to GFP (AtRGP2:GFP) in transgenic *N. tabacum* resulted in impaired TMV intercellular movement and enhanced callose accumulation at PD [193], while silencing *N. benthamiana* RGPs using virus-induced gene silencing resulted in enhanced TMV MP local spread and TMV systemic movement [194].

In another recent study, TMV MP was found to interfere with callose deposition at PD triggered by double-stranded RNA (dsRNA) through pattern-triggered immunity (PTI) [195]. The reduction of callose deposition at PD was more significant in cells at the TMV infection front compared to cells ahead of and behind the infection front. This shows that the virus inhibits callose deposition at PD in regions where unobstructed PD trafficking is necessary for the spread of the infection. The capacity to suppress dsRNA-induced callose deposition to evade this host defense mechanism was attributed to the TMV MP itself. Suppression was observed when MP was expressed ectopically without TMV infection but not when it was mutated to lose PD targeting and support virus movement (a P to S substitution at amino acid position 81). ORMV and TVCV MPs were found to function similarly to TMV MP in inhibiting dsRNA-induced callose deposition, suggesting a common strategy used by different tobamoviruses [195]. The SOMATIC EMBRYOGENESIS RECEPTOR-LIKE KINASE 1, the BOTRYTIS INDUCED KINASE1/AVRPPHB SUSCEPTIBLE1-LIKE KINASE1 kinase module, CALMODULIN-LIKE 41, calcium, and potentially PDLP1/2/3 were identified as components of the dsRNA-induced PTI signaling pathway, inducing callose deposition and antiviral immunity. It remains to be shown whether the MPs of TMV, ORMV, and TVCV interfere with dsRNA-induced callose deposition at PD through direct interactions with these components or by interacting with the callose turnover machinery at PD [195]. In summary, TMV MP modulation of callose deposition at PD to facilitate TMV intercellular movement is evident, but further investigation is still needed to fully understand the underlying mechanism.

Regarding MP involvement in membrane restructuring, specific members of a family of host proteins that shape the ER, the reticulons, interact with TMV MP [196]. Reticulons (RTNs) are integral ER membrane proteins that function in ER tubulation and can squeeze ER tubules to limit lumen flow [197]. Reticulons 3 (RTN3) and 6 (RTN6) localize to the PD, a finding that raises the possibility of a role in constricting the desmotubule to regulate PD permeability [198,199]. Interestingly, RTN3 and RTN6 interact with the ER-PM contact site proteins SYTA, SYT7, and VAP27 [182], and TMV MP, among other viral MPs, interacts with both RTN3 and 6 *in planta* [196]. Recently, SYT protein family members, SYTA, SYT5, and SYT7, located in SYTA-labelled ER-PM contact sites (S-EPCSs) developed around and in the PD were shown to aid ER-desmotubule squeezing at the PD entrances and within to sustain cytoplasmic sleeves [174]. An Arabidopsis *syt1 syt5 syt7* triple mutant displayed a detached cortical ER from the PM and diminished constriction of the ER at PD entrances (ER-desmotubule junctions). These findings suggest that S-EPCSs are involved in transforming the ER into desmotubules at the PD entrances. Moreover, in the *syt* triple mutant plants, the subcellular distribution of a YoMV MP-GFP fusion was altered and virus intercellular movement was inhibited. Strikingly, no effect on free GFP passive intercellular movement in these mutant plants was detected, suggesting that S-EPCSs function in constricting desmotubules to maintain open cytoplasmic sleeves and only enable the active transport of lipids or membrane-associated proteins, such as viral MPs [174]. All these findings point to a probable role of RTNs and EPCSs in constricting the desmotubule and facilitating TMV intercellular transport. Overall, we can speculate that TMV-MP interaction with RTNs and EPCSs could contribute to the constriction of the desmotubule, which would lead to enlarged PD cytoplasmic sleeves and facilitate active transport of desmotubule membrane- and/or vesicle-associated TMV vRNPs (Figure 3C,D).

Another group of host proteins utilized by TMV MP to modulate the plasmodesmal SEL consists of plant remorins (REMs). These plant proteins are predominantly located in nanodomains in the cytosolic leaflet of the PM lipid bilayer, both around the cell and in the PD, and are important during responses to biotic and abiotic stresses ([200]; for review see [201]). The *S. tuberosum* REMORIN of Group 1 isoform 3 (StREM1.3) regulates PD permeability and interferes with the gating activity of MPs of different plant viruses, including TMV [202,203]. The mechanism underlying REM-based PD apertures control is attributed to REM association with PD and modulating callose deposition at PD pitfields that leads to restriction of virus intercellular movement [203]. In *N. benthamiana,* transient overexpression of REM1.5 (NbREM1.5) inhibited TMV intercellular movement [204]. In addition, NbREM1.5 was found to interact with TMV MP *in planta*, and NbREM1.5 overexpression in *N. benthamiana* leaves resulted in callose accumulation at PD, reducing PD permeability. These findings suggest that NbREM1.5 interferes with the MP’s ability to increase PD SEL [204]. Moreover, using point mutant proteins, palmitoylation of NbREM1.5 was found to be essential for its maximum accumulation, PM localization, and ability to inhibit TMV-GFP intercellular spread [204]. In contrast to the role of REM proteins in restricting the intercellular movement of plant viruses, overexpression of NbREMs 1.1, 1.2, and 1.8 did not inhibit TMV intercellular movement [204]. Additionally, overexpression of *N. tabacum* cv. Samsun NN REM1.2 (Nt(sNN)REM1.2) enhanced intercellular movement of ToMV [205]. The MP of ToMV interacted with and induced aggregation of the otherwise uniformly distributed Nt(sNN)REM1.2 on the PM [205]. These findings indicate that the effect of individual family members within the Nb and Nt REMs on TMV spread may depend on just a few differences in the protein sequence of these closely related family members.

Pectin methylesterases (PMEs) are examples of proteins that positively regulate TMV trafficking through PD. PMEs are cell wall- and PD-associated enzymes that function in cell wall remodeling. They possess methyl esterase activity that digests pectin and releases methanol gas [206,207]. TMV and TVCV MPs interact with tobacco PME [208,209], and disruption of the TMV MP-PME interaction inhibits TMV intercellular movement [209]. Moreover, *N. tabacum* transgenic plants that silence PME [209] or ectopically express *Actinidia chinensis* pectin methylesterase inhibitor protein in tobacco [210] resulted in, respectively, delayed TMV systemic movement or reduced TMV intercellular movement and systemic spread. Additionally, PME is expressed in response to leaf damage, leading to increased methanol production and the induction of methanol-inducible genes (MIGs) such as BG, NbMIG-21 (which encodes a nucleolus-localized protein), and aldose-1-epimerase-like protein (NbAELP, previously known as NCAPP). The expression of these MIGs results in enhanced PD trafficking of tandemly fused GFP (2xGFP) and improved TMV intercellular spread and accumulation [211,212].

Calreticulins are chaperones and Ca^+2^-sequestering proteins that reside mostly in the ER lumen and function in protein folding and calcium ion homeostasis and signaling [213]. Calreticulins are also located in PD [213]. TMV MP interacts with calreticulin from different plant species in vitro and in vivo and colocalizes with it at the PD [214]. Ectopically expressed TMV MP-GFP in *N. benthamiana* transgenic plants overexpressing maize calreticulin showed MP-GFP association with MTs rather than PD and inhibited intercellular transport [214]. Whether calreticulins play a role in modulating PD permeability through their interaction with TMV MP, regulation of calcium ion homeostasis, or both remains to be determined. Changes in cytoplasmic calcium ion levels can modulate PD permeability. For example, injecting calcium into *Zea mays* cells induces a very rapid closure of PD, followed by reopening within 10 s, a response that cannot be explained by the slower, callose-dependent changes in PD permeability [215]. These findings may explain an old finding that treating root meristematic cells with EDTA, presumably chelating calcium ions, significantly promoted TMV infection [216].

Another host factor that promotes TMV intercellular movement is the tobacco transcription factor NTH201, which is a class II KNOX protein [217]. The cellular functions of class II KNOX proteins are not fully understood. However, recent findings have uncovered a role for class II KNOX transcription factors in regulating plant secondary cell wall formation [218]. NTH201 was found to colocalize, but not interact, with TMV MP at the PD, and overexpression and silencing experiments demonstrated its role in enhancing TMV MP accumulation, VRC formation, and TMV local spread [217]. The mechanism underlying the role of NTH201 in TMV infection was not clear due to the lack of understanding of the cellular functions of class II KNOX transcription factors at that time. However, it was speculated that the enhanced TMV intercellular movement was due to the accumulation of MP at early infection stages, indirectly facilitated by NTH201 [217]. Although NTH201 was not found to interact with TMV MP, a tobacco DnaJ-like protein, NtMPIP1, was found to interact with both NTH201 and TMV MP in a yeast three-hybrid assay [219]. TMV MP interacts with the substrate binding domain of NtMPIP1. Knocking down NtMPIP1 inhibited TMV intercellular movement, suggesting that a TMV MP-NtMPIP1-NTH201 protein complex might play a promotive role in TMV local spread. Moreover, a group of NbMIP1s were found to interact with TMV and ToMV MPs and facilitate TMV intercellular and systemic movement [220]. The NbMIP1s proteins are probably different from NtMPIP1, as they share limited amino acid identity (about 24%) and were suggested to help TMV infection by folding and stabilizing TMV MP, likely through recruiting the MP to Hsp70. Indeed, the DnaJ proteins are co-chaperones that interact with and assist Hsp70 chaperone proteins in protein folding, among other functions [221]. Notably, silencing Hsp70 in *N. benthamiana* plants reduced the size and number of TMV-GFP lesions and inhibited systemic movement, suggesting a promoting role for Hsp70 in TMV infection [222]. As noted earlier, Hsp70 was shown to co-purify with the TMV replication proteins [28]. In an earlier model for virus intercellular movement, Hsp70 proteins, known to traffic through PD [223], were proposed to actively transport vRNPs through PD [69]. Overall, an in-depth investigation is needed to understand the mechanisms underlying Hsp70/DnaJ proteins’ involvement in TMV trafficking through PD.

### 4.4. MP Phosphorylation

Several studies have shown that TMV MP is phosphorylated at different amino acid residues. The TMV MP phosphorylation appears to regulate its interaction with the vRNA, gating the PD, and supporting TMV intercellular spread. The TMV MP C-terminal phosphorylation at the Ser258, Thr261, and Ser265 amino acid residues by a cell wall-associated or plasmodesmal-associated protein kinase (PAPK) has been described [224,225,226]. Mimicking phosphorylation by amino acid substitutions at all three residues negatively impacted the ability of TMV MP to increase the plasmodesmal SEL and hence suppressed TMV intercellular movement [225]. Interestingly, this effect seems to be host-dependent, occurring in *N. tabacum* but not in *N. benthamiana*, *N. clevelandii*, or *N. glutinosa* [225,227], indicating that the inhibition of MP gating activity by C-terminal phosphorylation is adopted by one or a few hosts. In *N. tabacum*, the MP C-terminal phosphorylation was suggested to occur sequentially, as mimicking phosphorylation at a single site (e.g., Ser258 or Ser265) enhanced TMV intercellular movement, while mimicking phosphorylation at two or three sites inactivated the MP transport function, leading to reduced virus spread. Moreover, mimicking phosphorylation at Ser258 or Ser265 appeared to reduce MP association with ER and enhance MP accumulation at the PD, thus correlating with enhanced intercellular movement. In contrast, mimicking phosphorylation at Thr261 and Ser265 decreased the targeting of MP to PD and increased its association with MTs, which could lead to MP degradation and reduced movement [227]. In a later study analyzing the C-terminal phosphorylation of MP, it was evident that the MP is subjected to C-terminal phosphorylation early in infection at the ER before reaching the PD [228].

In addition to the C-terminal phosphorylation of the MP, phosphorylation of Thr104 by ER-associated kinases in vitro has been reported [229]. Mimicking phosphorylation at Thr104 using aspartate substitution appeared to inactivate the MP and strongly inhibited TMV intercellular movement in *N. tabacum* Xanthi nc. Other potential sites for MP phosphorylation have been identified, but their roles in TMV intercellular movement have not been characterized [230]. Overall, these studies on MP phosphorylation indicate that MP phosphorylation might be beneficial for both the host and TMV. Inactivation of MP transport functions by host-mediated phosphorylation would limit detrimental effects of a sustained increase in plasmodesmal SEL on cellular activities. However, MP phosphorylation within PD might be a mechanism used by TMV to switch from intercellular transport of its RNA genome to vRNA translation and replication once moved to new cells [231]. This hypothesis originated with the finding that unphosphorylated TMV MP, upon binding vRNA, renders it nontranslatable [231], while phosphorylated MP loses its ability to inhibit translation [232]. In living tissue, a complex of unphosphorylated TMV MP with vRNA infected intact plants but not protoplasts, which have no PD, while a complex of phosphorylated MP with vRNA infected both, suggesting the MP is being phosphorylated in the PD [232].

### 4.5. Cytoskeletal Proteins

The importance of the cytoskeletal elements in TMV intracellular movement has been discussed in detail earlier. Here we only will present findings that specifically may be associated with the PD or PD areas. Regarding MTs and intercellular TMV movement, MT function within PD is unlikely as MTs have not been observed in PD [233], and PD permeability as measured by electrical conductance was not affected by MT perturbation [234]. In contrast, actin filaments were speculated to be involved in TMV movement at PD due to the earlier observations that actin is present at the PD [235] and TMV MP can interact with actin in vitro [134]. Indeed, plasmodesmal orifice size and SEL were shown to increase when cells were treated with drugs that disrupt actin filaments, such as cytochalasin B and D, or profilin, a specific actin-binding protein [236,237]. Also, MFs affect PD permeability as inferred by PD electrical conductance [234]. Moreover, myosin associates with PD, and treating cells with 2,3-butanedione monoxime, an inhibitor of myosin, narrows the PD neck regions [238]. Regarding TMV and actin at the PD, actin filament severing activity demonstrated for TMV MP or CMV MP was associated with their ability to increase plasmodesmal SEL [239]. Treating cells with the actin-stabilizing agent phalloidin suppresses the increase in plasmodesmal SEL induced by TMV and CMV MPs, suggesting the importance of actin depolymerization to open PD. In agreement with the role of actin filaments in regulating PD permeability, Arabidopsis formin 2 (AtFH2), which functions in capping, stabilizing, and anchoring actin filaments to the PM at PD, was shown to regulate intercellular trafficking through PD [240]. Specifically, in *AtFH2* knockout mutants compared with wild-type plants, the SEL of PD is increased and was associated with greater intercellular trafficking of free GFP and CMV MP-GFP. Actomyosin activity may also be important in transporting vRNA to PD from adjacent areas. Specifically, both MFs and myosin class VIII members, 1, 2, and B, are important for locating MP-GFP to PD (e.g., [108,113]), and it was suggested that the class VIII myosins function to transport either an MP-vRNA complex alone or the entire VRC, directed by the internal MP-vRNA interaction, from cMERSs or other cortical sites to the PD [63,108,139].

### 4.6. MP Homeostasis

Regulating TMV MP cellular levels or homeostasis during infections, likely to protect cellular activities, has been studied in some detail in the 2000s. The MP of ORMV was found to complement MP-deficient TMV for intercellular movement at a faster rate than its own MP [33]. This was attributed to the lower accumulation levels of ORMV MP compared to TMV MP, suggesting an inhibitory role of MP overaccumulation in TMV intercellular movement. Therefore, a mechanism for spatially- and temporally-controlled MP degradation was investigated. One such mechanism could involve MP association to MTs. When expressed in cells, as noted in previous sections, TMV MP associates with ER, MTs, and PD [79,81,134]. Among these associations, our knowledge of the function of the MT association has evolved perhaps the most through the last 25 years. Earlier work considered the possibility of active MT-mediated transport of MP associated with vRNA to the PD (see previous sections on “intracellular movement”), but now there is growing evidence that MP-MT interactions are important for spatial positioning of the VRCs near the PD in cMERSs and for regulating the level of MP later in infection. Regarding the latter, Gillespie and co-workers determined that a TMV MP mutant, MP^R3^, with a diminished affinity for MTs, enhanced its own as well as infectious virus intercellular spread compared with wild-type MP [66]. They associated this enhanced spread with an inability of MP^R3^ to be degraded through a proteasome-mediated pathway, as has been suggested for wild-type TMV MP [65] (Figure 2B), and suggested that MTs were associated with a degradation process. Later, TMV MP was found to interact with an MT-associated protein from tobacco, MPB2C, that together accumulate at MTs [241]. MPB2C overexpression inhibited ectopically expressed MP intercellular movement. Silencing MPB2C in *N. benthamiana* led to loss of MP association with MTs but no change in virus spread, again leading to the suggestion that MTs were involved in something besides TMV spread [242]. The concept of MP homeostasis during TMV infection received strong support when it was determined that TMV MP induces and interacts with the host CDC48 [64]. CDC48 is an AAA+ ATPase (ATPase associated with various cellular activities) that functions as a segregase and unfoldase, playing an essential role in the ER-associated degradation pathway [243]. In this pathway, CDC48 works with other factors to segregate and translocate ubiquitinated, misfolded ER lumen and membrane proteins into the cytosol, where they can be refolded and recycled or transferred to the proteasome for degradation. The MP-CDC48 interaction results in translocation of the MP from the ER to the cytosol and increased MP association with MTs and its degradation [64]. Moreover, overexpressing CDC48 limits TMV intercellular movement, possibly due to interfering with the essential MP-ER association. This result is similar to the finding that overexpression of the MT and MP binding protein, MPB2C, decreased intercellular movement of TMV MP [241] in that MP association with MTs is associated not with intercellular trafficking of virus or virus components but with controlling MP accumulation. Recently, CDC48 was recognized for its important role in plant immunity, as tobacco plants overexpressing CDC48 increased salicylic acid accumulation in the leaves, leading to resistance to TMV infection [244].

### 4.7. Replication Proteins

As discussed earlier, TMV replication proteins, 126 kDa and 183 kDa, bind TMV RNAs, and as determined in genetic studies, the 183 kDa protein is sufficient for, and the 126 kDa protein may enhance, intercellular movement of the virus (for review see [28,61]). However, the method by which they support intercellular movement remains a significant mystery. Specifically, as noted, there is no evidence that: (a) the replication proteins associate with PD or move between cells, (b) the replication proteins and MP interact, or (c) a TMV genome expressing only the replication proteins moves cell to cell (see Section 3.1). Additionally, generational virus selection for movement showed a correlation between enhanced movement and mutations in the 126 kDa protein open reading frame [245]. The expression of functional replication proteins in the absence of MP and CP potentially triggers a stress response and callose deposition at the PD, resulting in impaired cell-to-cell transport of ER membrane proteins and cytosolic GFP [110]. This effect of callose deposition at PD and the reduced PD transport was reversed when the virus expressing only the replication proteins was inoculated into plants expressing TMV MP, suggesting that both replication proteins and MP are required to decrease callose deposition and facilitate PD trafficking. Based on these findings, it was proposed that TMV MP and replication proteins, in a complex with vRNA, move through PD by diffusion along the desmotubule membrane [110]. Perhaps related to this point and as noted earlier, TMV replication proteins interact with the chaperone, Hsp70, which is known to traffic through PD [223] and is proposed to actively transport vRNPs through PD [69].

## 5. Conclusions and Outlook

TMV has been at the forefront of plant virus research, studying mechanisms of disassembly, replication, and intra- and intercellular movement (e.g., [20,24,25,28,63,92]). While some advances in early cell biological and biochemical studies were made decades ago, they are still relevant and fundamental. An example of this is the elegant work showing in vivo disassembly of the TMV capsid, first exposing the 5’ end of the viral RNA for further co-translational disassembly as the replication proteins are synthesized and secondly exposing the 3’ end of the viral RNA for co-replicational disassembly (reviewed in [20]). These findings remain important in understanding the host-adapted replication strategy, as co-translational disassembly has been suggested to protect the vRNA as long as possible from host-mediated RNA degradation [20]. The demonstration of the association of the 126/183 kDa proteins and MP with the ER and cytoskeleton has been useful for those studying the roles of these host components in the accumulation and spread of other viruses [15,63]. TMV also stood out as the pioneer model virus in studies to identify viral products necessary for PD-mediated intercellular transport, as, for example, the use of TMV MP as a model for the study of MPs from other viruses. In addition, studies determining that other virus proteins, such as the 126/183 kDa protein(s), could impact this movement activity possibly through intracellular transport of VRCs and subsequent alteration of membrane activity through the PD, have served the greater virology community.

TMV is certainly a leading virus for which viral and host components involved in the infection process have been identified and studied. That said, there are many steps within this process that are not fully understood. Perhaps the most important finding in need of explanation for TMV, as well as other tobamoviruses, is the probable interplay between the ER membrane and vacuole/prevacuole membranes in replication and intracellular movement. Regarding intercellular tobamovirus movement, the persistent need to identify and determine the function of host and viral components near or at the PD remains critical.

Addressing unanswered questions on virus-host interactions requires the use of both existing and emerging technologies. For example, protein-protein interaction technologies, such as yeast two-hybrid and co-immunoprecipitation assays, have been powerful tools for investigating stable interactions involved in infection. However, the number of proteins that can be detected can be limiting, and newer technologies that help access the full potential of interactions should be utilized. In a recent study, a proximity-dependent biotin identification (BioID) method was used to identify transient, weak, or even proximal indirect protein interactions in vivo [246]. These researchers determined that multiple host proteins interact with the TMV 126 kDa protein, and altering the gene expression level of one of the interactors, NbSGT1, affected TMV infection. Importantly, this protein did not interact with the 126 kDa protein in a yeast two-hybrid assay. Gene editing, especially useful in plant hosts without a large mutant knockout collection, can be used to verify the importance of a host protein identified through BioID or other protein-protein interaction studies for TMV accumulation and spread. In cell biology research, significant advances in super-resolution optical microscopy and electron microscopy should help resolve several outstanding questions regarding the composition and dynamics of TMV VRCs. For example, recently, SARS-CoV-2 replication organelles, RNA, and proteins were imaged and characterized at the nanoscale level using super-resolution fluorescence microscopy [247]. Lastly, findings from studies on other viruses should always be reviewed in order to incorporate them into TMV research. Recently it was determined that amphipathic helices from different plant viral proteins could differentially bind specific membranes [248]. Amphipathic helices with essentially no sequence identity from the 1a replication proteins of BMV or CMV, fused with GFP, were observed, respectively, at the perinuclear ER membrane or tonoplast. These viruses were known to replicate using, respectively, ER membrane or the tonoplast, and therefore this specificity may be driven by the individual amphipathic helices. It will be important to study the predicted amphipathic helix in the TMV 126 kDa protein [78] to determine its influence in associating with ER membrane or vacuolar/prevacuolar membranes for virus accumulation and spread.
